# Physical and functional interaction between the HTLV-1 Tax oncoprotein and the cellular LIM domain protein FHL3

**DOI:** 10.1186/1742-4690-8-S1-A160

**Published:** 2011-06-06

**Authors:** Áine McCabe, William W Hall, Noreen Sheehy

**Affiliations:** 1 Centre for Research in Infectious Diseases, School of Medicine and Medical Science, University College Dublin, Dublin, Ireland

## 

HTLV-1 is the ethiologic agent of adult T-cell leukemia (ATL). The viral regulatory protein Tax1 plays a pivotal role in T-cell transformation by deregulating several signalling pathways including CREB, NF-κB and AP-1 leading to the abnormal expression of several cellular proteins involved in cell survival and proliferation. Previous studies in the lab, using the yeast-2-hybrid approach to screen a T-cell library for Tax1 interacting partners identified the cellular Four and a Half Lim domain protein 3 (FHL3) as a possible Tax1 interacting candidate. FHL3 can regulate the actin-cytoskeleton and functions as a transcriptional activator. The aim of this study is to investigate the physical and functional interaction between Tax1 and FHL3. We have found that Tax1 and FHL3 interact both in vitro and in vivo and deletion analysis of FHL3 revealed that each LIM domain of FHL3 can interact with Tax1. Deletion analysis of Tax1 determined that the interaction with FHL3 occurs at both the N and C terminus, namely amino acids 1-50 and a fragment which encompasses the predicted LIM binding domain 207-353aa. We have demonstrated that FHL3 enhances Tax1 mediated LTR transcriptional activation without affecting basal activity. In contrast to this we show that FHL3 down regulates NF-κB activation by Tax1. Using confocal microscopy, we show that FHL3 co localizes with Tax in the nucleus and at the periphery of co transfected cells. In addition we demonstrate that FHL3 induces nanotubules in Cos7 cells and may be capable of transporting Tax1 from one cell to another. Overall our results so far suggest that the interaction between Tax1 and FHL3 alters both the transactivating activity and the sub cellular localization of Tax1 and provide new insights into molecular mechanisms that underlie the oncogenic nature of this HTLV-1 protein.

